# The Characterization and Pathogenicity of a Recombinant Porcine Epidemic Diarrhea Virus Variant ECQ1

**DOI:** 10.3390/v15071492

**Published:** 2023-06-30

**Authors:** Xiaowei Mei, Jiahui Guo, Puxian Fang, Jun Ma, Mingxiang Li, Liurong Fang

**Affiliations:** 1 State Key Laboratory of Agricultural Microbiology, College of Veterinary Medicine, Huazhong Agricultural University, Wuhan 430070, China; meixiaowei@webmail.hzau.edu.cn (X.M.); guojiahui@webmail.hzau.edu.cn (J.G.); pxfang@mail.hzau.edu.cn (P.F.); ma.jun@webmail.hzau.edu.cn (J.M.); liveter@webmail.hzau.edu.cn (M.L.); 2 The Key Laboratory of Preventive Veterinary Medicine in Hubei Province, Cooperative Innovation Center for Sustainable Pig Production, Wuhan 430070, China

**Keywords:** porcine epidemic diarrhea virus, epidemiology, recombination, variant, pathogenicity

## Abstract

Porcine epidemic diarrhea virus (PEDV), a re-emerging enteropathogenic coronavirus, has become the predominant causative agent of lethal diarrhea in piglets, resulting in huge economic losses in many countries. Furthermore, the rapid variability of this virus has increased the emergence of novel variants with different pathogenicities. In this study, 633 fecal samples collected from diarrheic piglets in China during 2017–2019 were analyzed, and 50.08% (317/633) of these samples were PEDV-positive. The full-length spike (S) genes of 36 samples were sequenced, and a genetic evolution analysis was performed. The results showed that thirty S genes belonged to the GII-a genotype and six S genes belonged to the GII-b genotype. From the PEDV-positive samples, one strain, designated ECQ1, was successfully isolated, and its full-length genome sequence was determined. Interestingly, ECQ1 is a recombinant PEDV between the GII-a (major parent) and GII-b (minor parent) strains, with recombination occurring in the S2 domain of the S gene. The pathogenicity of ECQ1 was assessed in 5-day-old piglets and compared with that of the strain EHuB2, a representative of GII-a PEDV. Although both PEDV strains induced similar fecal viral shedding in the infected piglets, ECQ1 exhibited lower pathogenicity than did EHuB2, as evidenced by reduced mortality and less severe pathological changes in the intestines. These data suggest that PEDV strain ECQ1 is a potential live virus vaccine candidate against porcine epidemic diarrhea.

## 1. Introduction

Porcine epidemic diarrhea virus (PEDV) infection causes severe diarrhea, vomiting and dehydration with high mortality in neonatal piglets, leading to tremendous economic losses in the swine industry [1,2,3]. Porcine epidemic diarrhea (PED) was first reported in the United Kingdom in 1971 [4] and was then detected in numerous other swine-producing countries in Europe during the 1970s [5,6,7]. Since then, it has been transmitted to Asian countries, including China, South Korea, Thailand and Japan [8,9,10,11,12]. PED was not prevalent on a large scale worldwide until the emergence of a new PEDV variant in China at the end of 2010 [13,14,15,16]. The new PEDV variants that resulted caused not only high mortality in newborn piglets but also high morbidity in all age groups [17].To date, the prevention and treatment of PEDV infection have remained huge challenges [18]. The phylogenetic tree of full PEDV genomes shows that PEDV strains have evolved into two separate genogroups, GI (classical) and GII (variant), which are subdivided into five subgroups (GI-a, GI-b, GII-a, GII-b and GII-c) [19]. GII-c is also known as the S-INDEL subgroup.

PEDV belongs to the *Alphacoronavirus* genus within the *Coronaviridae* family. It is an enveloped, single-stranded positive-sense RNA virus with a full-length genome of approximately 28 kb [20,21,22]. Similar to other coronaviruses, the spike (S) protein of PEDV is heavily glycosylated and plays a crucial role in the virus entry, immunogenicity and pathogenicity. Extensive research efforts have led to the identification of various neutralizing epitopes within the spike proteins of the GI and GII strains of PEDV [23]. These epitopes hold significant potential as primary targets for the development of vaccines and therapeutic interventions [24,25]. The S gene of PEDV is considered an essential target gene for comprehending the genetic relationships and epidemiological profiles of field isolates, as well as for the development of new generations of vaccines [26,27].

Recombination greatly contributes to virus evolution, and recombination events are frequently observed in various viruses, particularly RNA viruses [28]. High-frequency RNA recombination may signify an unparalleled feature of coronavirus replication [29]. Recombination was principally observed in seven major genome-wide regions of PEDV, including non-structural protein 2 (nsp2), nsp3, nsp14-16, S1 and the nucleocapsid (N) gene [30]. Recombination enriches the genetic diversity of PEDV and continues to be a challenge in PEDV prevention and treatment [31].

Here, we report the emergence of a new natural recombination event, in which the ECQ1 strain was formed by the replacement of most of the S2 gene from the GII-b group of the PEDV 17GXCZ-1ORF3c-like strain with that from the highly pathogenic GII-a group strain (namely PEDV-1C-like) through natural recombination. The pathogenic features and antigen distribution of strain ECQ1 are described, along with its genetic and phylogenetic characterization and evidence of recombination events.

## 2. Materials and Methods

### 2.1. Ethics Statement

All procedures involving animal experiments were reviewed by, approved by and conducted in strict accordance with the Animal Experimental Ethical Inspection of Laboratory Animal Centre, Huazhong Agricultural University (Ethics Approval Number: HZAUSW-2023-0013).

### 2.2. Sample Collection and PEDV Detection

From July 2017 to November 2019, 633 samples comprising small intestinal tissues and feces were collected from diarrheic piglets across 19 provinces in China. The intestinal samples were processed by homogenization in phosphate-buffered saline (PBS) to obtain 10% suspensions, while the stool samples were diluted with PBS to achieve 10% suspensions. Subsequently, the suspensions were vortexed, then centrifuged at 1700× *g* at 4 °C for 10 min to gain clarified supernatants. Viral RNA extraction was performed using the TRIzol reagent, followed by cDNA synthesis using the TaKaRa RNA PCR Kit (AMV) Ver.3.0 (TaKaRa Biomedical Technology, Beijing, China), as per the manufacturer’s instructions. To detect PEDV in the samples, specific primer pairs targeting the N gene of PEDV (5′-GCAACAACAGGTCCAGAT-3′ and 5′-CTCACGAACAGCCACATT-3′) were used. The expected size of the RT-PCR product was 563 bp [32].

### 2.3. Amplification of the Complete S Gene and Whole Genome Sequence of PEDV

To obtain the complete S gene sequence, a subset of positive samples collected from pig farms underwent viral RNA extraction and RT-PCR analysis using primer pairs that have been previously described [33]. Highly positive cases of PEDV were subsequently chosen for amplification of the full-length S gene and DNA sequencing analysis. The entire genome sequence was determined using 33 pairs of primers, as has been described in a previous study [33]. The amplified DNA fragments were then subjected to DNA sequencing, and the resulting sequences were assembled using DNAMAN software.

### 2.4. Sequence Alignment and Phylogenetic Analyses

To conduct a comprehensive genome phylogenetic analysis, we incorporated 32 complete genome sequences of previously reported PEDV strains and two newly identified sequences from our laboratory, namely ECQ1 and EHuB2. We utilized a total of 68 S gene sequences for phylogenetic analysis, including 36 S gene sequences from clinical samples and 32 of the reference sequences mentioned earlier. Multiple sequence alignment analysis was performed using MAFFT v.7.402 [34]. The maximum-likelihood phylogenetic tree (ML tree) was constructed using IQ-TREE software [35], with bootstrap values calculated, for each node, from 1000 replicates. The resulting tree was visualized using the ChiPlot online tool accessed on 19 October 2022 (https://www.chiplot.online/).

### 2.5. Genome Recombination Event Analysis

An analysis of genome recombination events was conducted using the Recombination Detection Program (RDP, version 4.97) and SimPlot software (version 3.5.1). Initially, seven recombination detection methods (RDP, MaxChi, GENECONV, BootScan, Chimaera, SiScan and 3SEQ) available in the RDP4 software were employed to identify the recombination signals within the entire gene sequence. To ensure the reliability of the detection, confirmation of a presumed recombination event based on recombination breakpoints was identified with at least six of these methods (*p* value set at 0.01). Next, the nucleotide (nt) sequence similarity was assessed using SimPlot software [36]. A sliding window size of 500 base pairs, a step size of 100 nts and 1,000 bootstrap replicates were employed using gap-stripped alignments and the maximum-likelihood (ML) distance model. GraphPad Prism 8.0 software (GraphPad Software, Inc., La Jolla, CA, USA) was employed to present the results of the nucleotide (nt) sequence similarity analysis.

### 2.6. Viral Isolation, Purification and Propagation

Viral isolation, purification and propagation procedures were as follows. Vero cells (ATCC CCL-81) cultured in Dulbecco’s Modified Eagle’s Medium (DMEM) (Gibco) supplemented with 10% fetal bovine serum (FBS) were utilized for PEDV isolation. For PEDV propagation, the maintenance medium consisted of DMEM supplemented with 7.5 μg/mL trypsin. When the Vero cells reached 70–80% confluence in 6-well cell culture plates, they were washed three times with PBS. Subsequently, the cells were incubated with a diluted suspension of PEDV for adsorption at 37 °C for 2 h. Following incubation, the cells were washed three times with PBS. Then, 2 mL of a maintenance medium containing 7.5 μg/mL trypsin was added to each well. The plates were returned to a 37 °C incubator with 5% CO_2_ for several days. Upon observation of visible cytopathic effects in the cells, the plates underwent a freeze–thaw treatment twice. The resulting supernatants were stored at −80 °C as seed stocks for plaque purification and subsequent passages.

### 2.7. Growth Curve Assay

To construct the viral growth curve, Vero cells in 12-well plates were infected with PEDV at a multiplicity of infection (MOI) of 0.1. After incubation at 37 °C for 2 h, the inoculum was removed; the plates were washed with PBS three times and covered with fresh DMEM (supplemented with 7.5 μg/mL trypsin). The supernatant and cells were harvested at 6, 12, 18, 24, 30, 36, 42 and 48 h post-infection (hpi), respectively. Then, the viral titers of these harvested samples were determined with a 50% tissue culture infectious dose (TCID_50_) assay.

### 2.8. Immunofluorescence Assay

Vero cells seeded in 12-well plates were infected with PEDV at an MOI of 0.1 for 12 h, then fixed with 4% paraformaldehyde for 15 min and then permeabilized with cold methanol for 10 min at room temperature, followed by washing three times with PBS. After blocking with 5% bovine serum albumin for 1 h, the cells were washed three times with PBS and then incubated with monoclonal antibodies (dilution: ×500) against the PEDV N protein for 1 h at 37 °C, followed by fluorescein isothiocyanate (FITC)-conjugated goat anti-mouse IgG. The cell nuclei were counterstained with 0.01% 4′,6-diamidino-2-phenylindole (DAPI) for 15 min at room temperature. Finally, the cells were rinsed with PBS three times and subjected to fluorescence microscopy analysis.

### 2.9. Animal Experiment

Fifteen 5-day-old piglets were obtained from a pig farm in Hubei, China. Prior to this experiment, the piglets were tested with RT-PCR and confirmed to be negative for PEDV, porcine deltacoronavirus (PDCoV), transmissible gastroenteritis virus (TGEV) and porcine rotavirus (PRoV). In addition, the sows that farrowed these piglets were further determined to be seronegative for PEDV antibodies using a virus neutralizing (VN) test. The piglets were then randomly divided into three groups, with five piglets in each group. Two groups were inoculated with strains ECQ1 and EHuB2, respectively, and the third group was designated as the negative control. Separate rooms were provided for each group. In the two PEDV inoculation groups, the piglets were orally administered the PEDV isolate at a titer of 10^6.0^ TCID_50_/mL, with a volume of 2 mL per piglet. The control group received 2 mL of maintenance medium per piglet. Daily observations were made to monitor and record clinical signs such as diarrhea, vomiting, anorexia and depression. Rectal swabs were collected before inoculation and on days 1, 3, 5, 7 and 9 after a PEDV challenge for virus shedding detection with real-time reverse transcriptase quantitative PCR (RT-qPCR) [37]. At 48 hpi, one piglet randomly selected from each group was subjected to necropsy. During necropsy, samples of the duodenum, jejunum and ileum were collected for histopathology, immunohistochemistry and viral load analysis, as has been described previously [37,38].

### 2.10. Statistical Analysis

All statistical analyses were conducted using GraphPad Prism 8.0 software, and significance was assessed using Student’s *t*-test. Experimental data where *p* < 0.05 were considered statistically significant, and a value of *p* < 0.01 was considered extremely significant.

## 3. Results

### 3.1. Epidemiological Investigation of PEDV in China from 2017 to 2019

**Figure 1:**
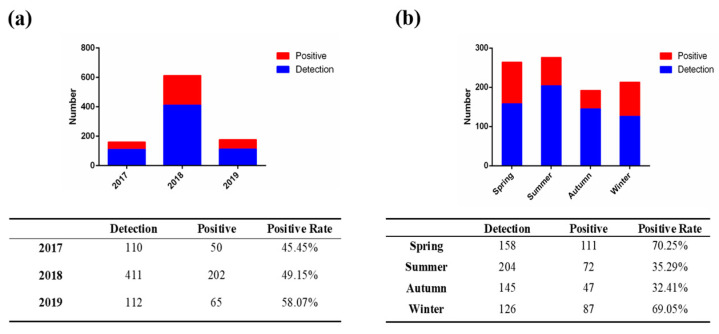
Detection of PEDV infection in piglets in 19 provinces of China. (**a**) The overall prevalence of PEDV infection in piglets with diarrhea from 2017 to 2019. (**b**) The seasonal prevalence of PEDV infection in piglets with diarrhea from 2017 to 2019.

To understand the molecular epidemiology and evolutionary diversity of the main swine enteric coronaviruses, including PEDV, TGEV and PDCoV, we collected 633 fecal samples of diarrheic piglets in 19 provinces during 2017–2019 (Table S1) and performed RT-PCR analysis. As shown in Figure 1a, PEDV was found to be the dominant cause of diarrhea in piglets, with an average positive detection rate of 50.08%. The positive detection rate of PEDV showed a sustained increase and reached 58.07% in 2019. Notably, 12.30% of PEDV-positive samples were co-infected with PDCoV, followed by 2.84% with TGEV, suggesting that PEDV–PDCoV co-infection was the dominant type of co-infection in PEDV-positive samples. Furthermore, the positive detection rate of PEDV was significantly higher in spring (70.25%) and winter (69.05%) than in summer (35.29%) and autumn (32.41%), indicating that outbreaks of PEDV primarily occur in spring and winter (Figure 1b).

### 3.2. Phylogenetic Analysis of Full-Length S Genes of PEDV

**Figure 2:**
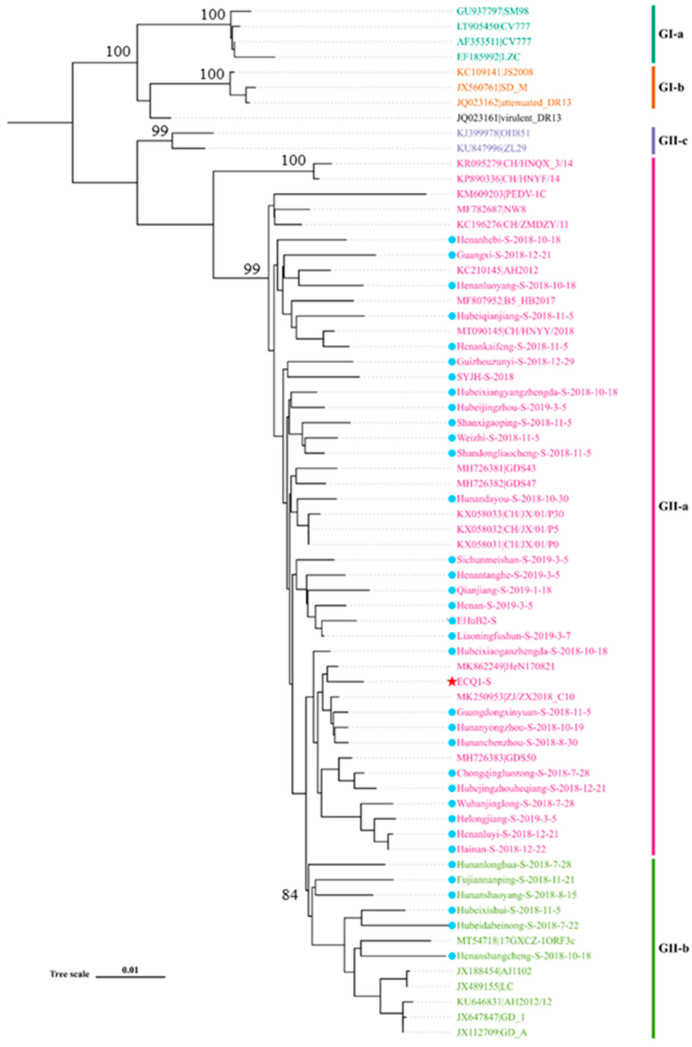
Phylogenetic tree analysis based on the S gene of PEDV. The maximum-likelihood phylogenetic tree (ML tree) was constructed using IQ-TREE software. The PEDV strains isolated in this study are indicated by red pentagons and blue circles.

To investigate the characteristics of the PEDV strains, we selected a subset of 36 PEDV-positive samples for full-length S gene sequencing (Supplementary Materials). A phylogenetic tree was constructed using the S gene sequences obtained from these 36 samples, along with the S gene sequences from 32 reference PEDV strains available in the GenBank database. The resulting phylogenetic tree, depicted in Figure 2, reveals the presence of two distinct genogroups: GI (classical) and GII (variant). All of the S gene sequences obtained in this study belong to the GII genogroup, with thirty sequences classified in the GII-a subgroup (e.g., AH2012 and B5-HB2017) and six sequences classified in the GII-b subgroup (e.g., AJ1102 and LC). These findings indicate that the majority of the epidemic PEDV strains in China in recent years have belonged to the GII genogroup, with the GII-a subgroup emerging as the dominant subgroup.

### 3.3. Isolation, Identification and Full-Length Genome Sequence and Phylogenetic Analyses of PEDV Strain ECQ1

**Figure 3:**
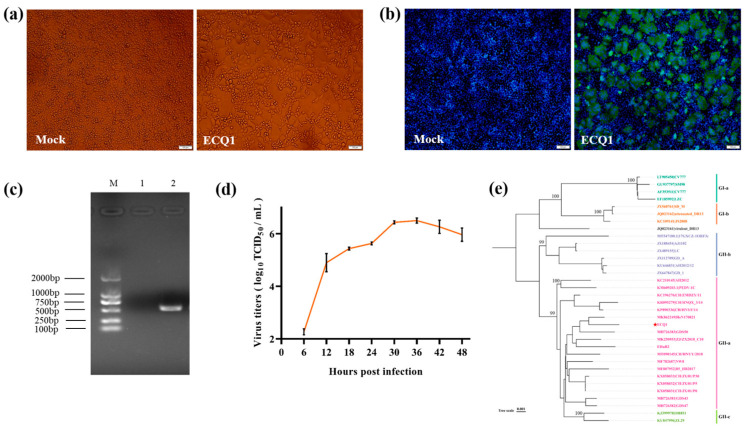
Isolation and identification of PEDV strain ECQ1 and phylogenetic tree analysis based on its complete genome. (**a**) Cytopathic effects were observed in PEDV ECQ1-infected Vero cells. (**b**) IFA for identifying ECQ1 in Vero cells using monoclonal antibodies against the PEDV N protein. (**c**) RT-PCR for amplifying an expected product size of 563 bp for the PEDV N gene detection. M: DL 2000 marker; Lane 1: negative control; Lane 2: ECQ1. (**d**) Determination of the growth kinetics of PEDV strain ECQ1 in Vero cells. Vero cells were inoculated with ECQ1 strains (MOI = 0.1). Cells were harvested at different time points post-infection (6, 12, 18, 24, 30, 36, 42 and 48 hpi) and then subjected to a TCID_50_ assay. (**e**) The maximum-likelihood phylogenetic tree was constructed using IQ-TREE software. The PEDV strain isolated in this study is indicated by red pentagons.

Initially, we conducted preliminary recombination analysis on the 36 sequenced S genes, revealing a recombination signal in the S gene of ECQ1. Consequently, our aim was to gain a comprehensive understanding of the complete genome and pathogenic characteristics of PEDV strain ECQ1. To achieve this, we isolated PEDV strain ECQ1 from an intestinal sample and performed complete genome sequencing. During the isolation process, notable CPEs, such as cell rounding, enlargement, detachment and syncytia formation, were observed in the cells following inoculation with the intestinal sample (Figure 3a). This PEDV isolate was then plaque-purified three times. Subsequently, the isolated PEDV strain, named ECQ1, was passaged in Vero cells for five generations and confirmed as PEDV-positive through an immunofluorescence assay (IFA) and RT-PCR (Figure 3b,c). Additionally, a growth curve of the ECQ1 isolate was generated based on the TCID_50_ values. As shown in Figure 3d, the titer of ECQ1 exhibited a gradual increase in Vero cells ranging from 6 to 36 hpi. The viral titer peaked at 36 hpi, with a mean titer of 3.16 × 10^6^/mL, followed by a gradual decline.

The full-length genome of ECQ1 was sequenced (GenBank accession number OQ412638) and compared with the genomes of representative PEDV strains. These comparative analyses showed that the nucleotide identities between ECQ1 and the GII variant strains (e.g., AJ1102, PEDV-1C, AH2012, OH851 and 17GXCZ-1ORF3C) ranged from 97.9% to 98.4%, while the nucleotide identities with the GI prototype strains (e.g., CV777, JS2008, SM98 and attenuated DR13) ranged from 95.9% to 96.6%. ECQ1 shared a >98.3% nucleotide identity with PEDV-1C in the ORF1a/1b, E, M, N and 3′ UTR regions but displayed lower nt identities (95.7%) in the ORF3 gene. The phylogenetic analyses based on the complete genome sequences showed that PEDV strain ECQ1 belongs to the GII-a subgroup, which includes the earlier GII-a PEDV strains found in China (e.g., AH2012 and PEDV-1C) and the EHuB2 strain isolated in our laboratory (Figure 3e).

### 3.4. Recombination Analysis of PEDV Strain ECQ1

**Figure 4:**
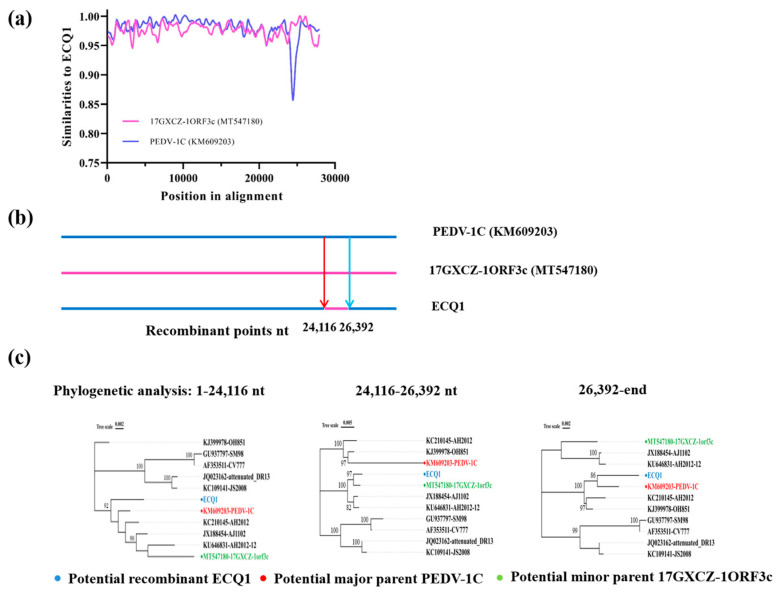
Recombination analysis of the complete genomic sequence of PEDV strain ECQ1. (**a**) Recombination detection program and SimPlot software were used to conduct the recombination analysis for the entire gene. Potential recombination events were identified based on strong *p* value (<10^−10^). (**b**) The PEDV ECQ1 genome exhibited recombination originating from two sources: 1. from positions 1 to 24,116 bp and 26,392 bp to the end of the PEDV-1C-like strain and 2. at positions 24,116–26,392 bp for the 17GXCZ-1ORF3c-like strain. (**c**) The maximum-likelihood phylogenetic tree was constructed using IQ-TREE software.

Recombination can lead to rapid changes in the genetic diversity of RNA viruses [39]. We used Recombination Detection Program version 4 (RDP4) software and SimPlot v.3.5.1 to determine the specific details of the ECQ1 strain recombination event. Six methods in RDP4 strongly supported that the ECQ1 strain is a natural recombinant virus with an almost identical S2 domain of the S gene to that of the 17GXCZ-1ORF3c-like strain, and the remaining genomic regions were from the PEDV-1C-like strain. The level of consensus among multiple methods was considered statistically significant (*p* value < 10^−10^). SimPlot analysis further confirmed the chimeric nature of the ECQ1 strain (Figure 4a). A statistically significant signal for phylogenetic incongruence was detected in the ECQ1 strain, defining two recombinant breakpoints in the PEDV genome: (1) from positions 1-24,116 bp and 26,392 bp to the end of the genome of the PEDV-1C-like strain and (2) at positions 24,116–26,392 bp of the 17GXCZ-1ORF3c-like strain near the S2 domain of the S gene of the ECQ1 strain (Figure 4b). A phylogenetic analysis of these fragments using the ML method also proposed the PEDV-1C-like strain as the major parent and the 17GXCZ-1ORF3c-like strain as the minor parent, providing strong evidence of recombination events (Figure 4c). Taken together, these findings demonstrate that the evolution of strain ECQ1 resulted from a recombination event, with acquisition of the S2 domain of the S gene from the 17GXCZ-1ORF3c-like strain and the remaining genomic regions originating from the PEDV-1C-like strain.

### 3.5. Pathogenicity of ECQ1 in Piglets

**Figure 5:**
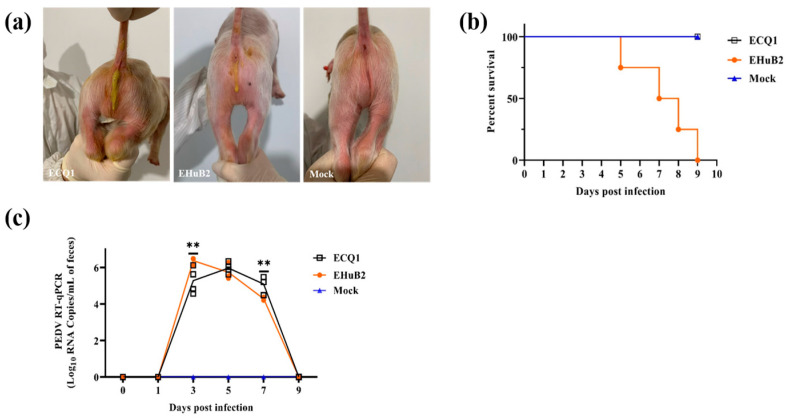
Pathogenicity of PEDV strain ECQ1 in piglets. (**a**) Clinical evaluation of piglets following PEDV challenge. Piglets, aged 5 days, were examined 2 days after inoculation with PEDV strains ECQ1 and EHuB2, with DMEM as a control. (**b**) Percentage of piglets that survived in each group. (**c**) Fecal excretion by 5-day-old piglets inoculated with different PEDV strains. ** *p* < 0.01, ECQ1 vs. EHuB2.

To assess the pathogenicity of PEDV strain ECQ1, we used EHuB2 (a non-recombinant GII-a strain) as a positive control and 5-day-old piglets were challenged with the same doses of PEDV strains ECQ1 and EHuB2. All piglets from the negative control groups were active and fleshy during this study, with no observed clinical signs. The piglets in the ECQ1 group began to show diarrhea at 1 day post-infection (DPI), while the piglets in the EHuB2 group developed diarrhea at 2 DPI, 24 h later than the piglets in the former group (Figure 5a). Lethargy, anorexia and diarrhea were the most severe in the challenged piglets at 1-4 DPI, and the piglets in the ECQ1 group gradually recovered thereafter (Table 1). However, more piglets in the EHuB2 group developed more severe symptoms of diarrhea, vomiting, lethargy and anorexia at 5–9 DPI (Table 1). Notably, after oral inoculation with PEDV-EHuB2, one piglet died at 5 DPI and all piglets died at 9 DPI, while no deaths were observed in the ECQ1 group (Figure 5b).

**Figure 6:**
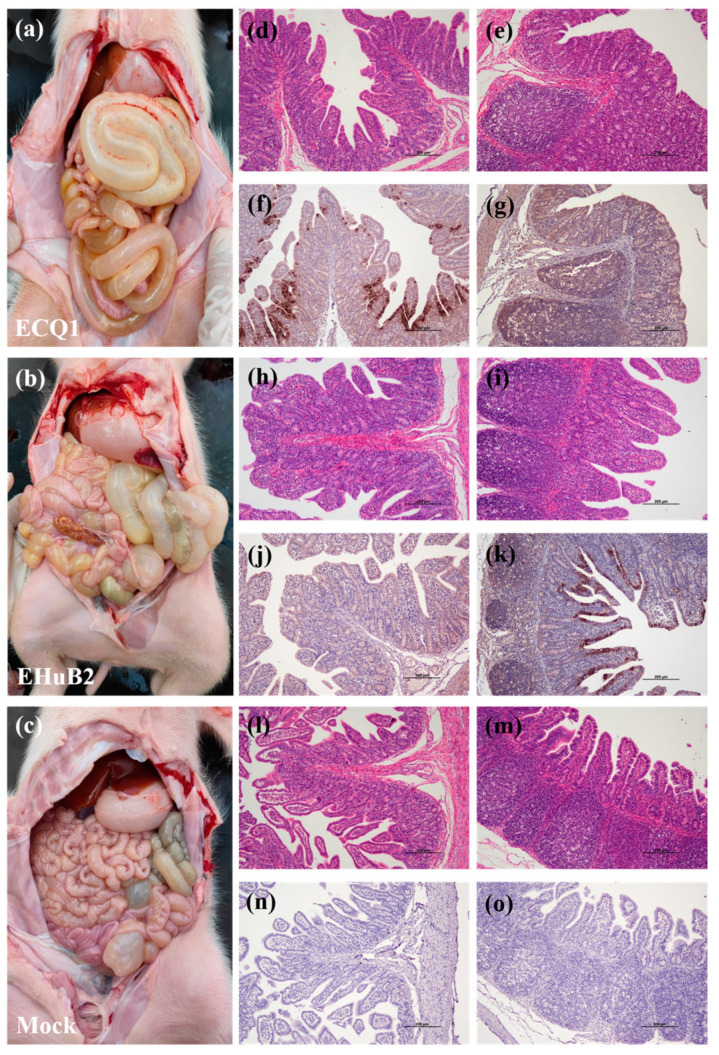
Intestinal lesions of PEDV-challenged piglets at 2 DPI. Macroscopic lesions found in piglets of ECQ1 (**a**), EHuB2 (**b**) and mock groups (**c**) in necropsy. H&E-stained jejunum (**d**,**h**,**l**) and ileum (**e**,**i**,**m**) tissue sections in the ECQ1 (**d**,**e**), EHuB2 (**h**,**i**) and mock groups (**l**,**m**). Immunohistochemically stained jejunum (**f**,**j**,**n**) and ileum (**g**,**k**,**o**) tissue sections in the ECQ1(**f**,**g**), EHuB2 (**j**,**k**) and mock groups (**n**,**o**).

We also compared fecal viral shedding and virus distribution between the PEDV-ECQ1- and PEDV-EHuB2-challenged piglets from 1 to 9 DPI with RT-qPCR. These results indicated that all piglets in the control group were negative for PEDV, while those in both the ECQ1 and EHuB2 groups exhibited high levels of virus shedding in the feces. The PEDV RNA copy numbers in the ECQ1 and EHuB2 groups were detected with the mean titers of 5.97 log10 copies/mL at 5 DPI and 6.38 log10 copies/mL at 3 DPI, respectively (Figure 5c). The virus distributions in different tissues were also tested in two 5-day-old piglets in necropsy at 2 DPI. Both the jejunum (7.31 log10 copies/g) and ileum (5.39 log10 copies/g) exhibited viral loads in the ECQ1 group, whereas only the ileum (7.29 log10 copies/g) exhibited viral loads in the EHuB2 group. The viral load in the ileum in the EHuB2 group was higher than that in the ECQ1 group. No viral load was detected in the duodenum of the ECQ1 or EHuB2 groups. Pathological examination revealed that the intestinal walls of the piglets in both the ECQ1 and EHuB2 groups were thin, transparent and gas-distended in the intestinal cavity (Figure 6a,b). No lesions were observed in any other organ of the PEDV-challenged piglets or in the organs in the negative control pigs in necropsy (Figure 6c). Using H&E staining, the infected piglets were characterized by shortening, atrophy or even shedding of intestinal villi (Figure 6d,e,h,i) compared with piglets from the negative control groups (Figure 6l,m). Consistently with the viral loads in different tissues, the PEDV antigen was detected with immunohistochemical staining in the ileum of the EHuB2 group (Figure 6k), whereas the antigen was detected in the jejunum of the ECQ1 group (Figure 6f).

## 4. Discussion

The first PED outbreak occurred in England in 1971, and to date, a great number of PEDV strains with diverse genetic, antigenic and pathologic characteristics have been described [8]. As one of the most devastating swine enteric diseases, PED has posed a tremendous threat to the swine industries of many countries in recent years, seriously impacting production and leading to huge economic losses. In this study, a high prevalence of infection with PEDV, 50.08%, was detected in clinical samples from pigs with diarrhea. The circulating strains were genetically classified into two groups, namely GI and GII, based on S-gene-sequence analysis. Only GII strains were detected in our samples collected during 2017–2019, suggesting that the classic GI strains were rarely circulating in China during that time. More detailed subgroup analysis showed that the 30 S gene sequences obtained belonged to the GII-a subgroup. The GII-a subgroup of PEDV is therefore the main pathogen causing PED in China at present.

As is known, the PEDV S protein plays a critical role in binding to cellular receptors to initiate infection and induces neutralizing antibodies in vivo [40]. Considerable genetic variations in the S gene between newly determined PEDV field strains and early isolates have been reported, and these variations may lead to changes in the pathogenicity and tissue tropism of PEDV [41]. As with other coronaviruses, the diversity of PEDV is also driven by genetic recombination [19]. Previous research has reported occurrences of recombination events in various PEDV strains [42]. A previous study has reported that the potential recombination breakpoints in the S-INDEL strain genome may be located between ORF 1a and ORF 1b, between S1 and S2, and between ORF 3 and E gene [43]. Another finding revealed that CH/HNQX-3/14 may be a mosaic strain resulting from a recombination event involving three putative parental-like strains (CV777, DR13 and CH/ZMDZY/11) [44]. Interestingly, we report a special recombinant strain, ECQ1, that resulted from the recombination of the S2 domain of the S gene from the GII-b 17GXCZ-1ORF3c-like strain with the remaining genes of the GII-a PEDV-1C-like strain. Most reports have indicated that recombination in PEDV is more likely to occur in the nsp2, nsp3, nsp14–16, S1 and N gene than in the S2 domain of the S gene [30]. Therefore, further understanding of the biological and virological characteristics of ECQ1 will help to expand our knowledge of PEDV.

The pathogenicities of different prototype strains have been assessed in neonatal piglets. It was recently reported that PEDV strain SH, which contains a consecutive 12 aa deletion, including an antigenic epitope, NEP-1C9, in the N protein, is highly pathogenic in suckling piglets [45]. Yan et al. reported that a GII-c subgroup strain, NH-TA2020, caused watery diarrhea within 24 hpi, indicating its strong pathogenicity [46]. In addition, it has been reported that pigs inoculated with the S-INDEL variant isolate exhibited significantly reduced clinical signs, virus shedding in feces, histopathological lesions in the small intestines and viral antigen in the ileum compared with pigs inoculated with the three US PEDV prototype isolates [41]. Although there is increasing evidence that PEDV routinely undergoes significant variation, particularly in the spike protein, the pathogenicities of PEDV strains with different variants of the S protein are not well defined. Here, ECQ1, a GII-a strain with natural recombination in the S2 domain of the S gene, was used to assess pathogenesis in 5-day-old piglets by oral inoculation. Piglets inoculated with EHuB2, a typical PEDV GII-a strain, had high viral antigen content in the ileum and 100% lethality, whereas piglets inoculated with ECQ1 had high viral antigen content in the jejunum and no lethality. These different results might be due to the necropsy of a single piglet per group, which may not have been representative. In addition, the dynamic progression of antigen distribution in the early stages of infection was not fully characterized in the present study. Regarding intestinal tropism, a study has shown that the novel PEDV variant TTR-2/JPN/2014, with a large deletion in the S gene, was non-lethal in highly susceptible neonatal piglets. Furthermore, an important region within the deletion of 582 nucleotides in the S gene was confirmed to be responsible for tissue invasion, directivity and virulence in newborn piglets [47,48,49,50]. The actual mechanisms involved in pathogenicity, tissue tropism and virulence should be related to factors other than the N-terminal sequence of the S protein [51]. These factors, such as changes in other genomic regions, alterations in viral protein structure or function, the genetic background of the piglets and the availability of functional receptors, fusion-activating proteases and alterations in cleavage requirements in different porcine inoculation models, should be taken into consideration [52]. A previous study has indicated that mutations in the S2 domain of coronaviruses have been linked to changes in tissue tropism [53]. These results suggest that the S2 domain might be associated with the pathogenicity of PEDV and potentially affects the tissue tropism of PEDV.

In this study, we identified a natural recombination event in the S2 domain of the S gene in PEDV strain ECQ1. This recombination event has the potential to impact the structure of the S protein, which could directly or indirectly influence viral tropism and pathogenicity, resulting in a decrease in viral virulence. Moreover, our findings indicate that the PEDV ECQ1 strain holds promise as a potential candidate for a modified live virus vaccine against PED. Furthermore, the emergence and evolution of these PEDV variants in the field require further investigation, as understanding these processes will significantly contribute to the global prevention and control of PED outbreaks.

## Figures and Tables

**Table 1:** Clinical observation records of 5-day-old piglets challenged with ECQ1 and EHuB2 strains.

DPI	Strain	Clinical Observation								Fecal Consistency		
		a	b	c	d	e	f	g	h	Normal	Mild Diarrhea	Water Diarrhea
0	ECQ1	√								5/5	0/5	0/5
	EHuB2	√								5/5	0/5	0/5
1	ECQ1		√							2/5	3/5	0/5
	EHuB2	√								5/5	0/5	0/5
2 *	ECQ1					√				2/4	2/4	0/4
	EHuB2						√			2/4	2/4	0/4
3	ECQ1				√					2/4	2/4	0/4
	EHuB2							√		1/4	3/4	0/4
4	ECQ1			√						3/4	1/4	0/4
	EHuB2								√	1/4	3/4	0/4
5	ECQ1	√								4/4	0/4	0/4
	EHuB2								√	0/3	1/3	2/3
6	ECQ1	√								4/4	0/4	0/4
	EHuB2								√	0/3	0/3	3/3
7	ECQ1	√								4/4	0/4	0/4
	EHuB2								√	0/2	0/2	2/2
8	ECQ1	√								4/4	0/4	0/4
	EHuB2								√	0/1	0/1	1/1
9	ECQ1	√								4/4	0/4	0/4
	EHuB2								√	0	0	0

* One piglet was necropsied at 2 days post-inoculation. (a) All active and eating well; (b) All active, 25% with vomiting and anorexia; (c) All lethargic, 25% with vomiting and anorexia; (d) All lethargic, 50% with vomiting and anorexia; (e) All lethargic, 75% with vomiting and anorexia; (f) 25% lethargic, 75% with vomiting and anorexia; (g) 50% with lethargy, vomiting and anorexia; (h) All with lethargy, vomiting and anorexia. √ indicates clinical symptoms.

## Data Availability

The datasets generated in this study are available upon request from the corresponding authors.

## Supplementary Materials

The following supporting information can be downloaded at: https://www.mdpi.com/article/10.3390/v15071492/s1.

## References

[1] Sueyoshi M., Tsuda T., Yamazaki K., Yoshida K., Nakazawa M., Sato K., Minami T., Iwashita K., Watanabe M., Suzuki Y. (1995). An immunohistochemical investigation of porcine epidemic diarrhoea. J. Comp. Pathol..

[2] Have P., Moving V., Svansson V., Uttenthal A., Bloch B. (1992). Coronavirus infection in mink (Mustela vison). Serological evidence of infection with a coronavirus related to transmissible gastroenteritis virus and porcine epidemic diarrhea virus. Vet. Microbiol..

[3] Debouck P., Pensaert M. (1980). Experimental infection of pigs with a new porcine enteric coronavirus, CV 777. Am. J. Vet. Res..

[4] Wood E.N. (1977). An apparently new syndrome of porcine epidemic diarrhoea. Vet. Rec..

[5] Song D., Park B. (2012). Porcine epidemic diarrhoea virus: A comprehensive review of molecular epidemiology, diagnosis, and vaccines. Virus Genes.

[6] Carvajal A., Lanza I., Diego R., Rubio P., Carmenes P. (1995). Evaluation of a blocking ELISA using monoclonal antibodies for the detection of porcine epidemic diarrhea virus and its antibodies. J. Vet. Diagn. Investig..

[7] Martelli P., Lavazza A., Nigrelli A.D., Merialdi G., Alborali L.G., Pensaert M.B. (2008). Epidemic of diarrhoea caused by porcine epidemic diarrhoea virus in Italy. Vet. Rec..

[8] Wang D., Fang L., Xiao S. (2016). Porcine epidemic diarrhea in China. Virus Res..

[9] Song D.S., Yang J.S., Oh J.S., Han J.H., Park B.K. (2003). Differentiation of a Vero cell adapted porcine epidemic diarrhea virus from Korean field strains by restriction fragment length polymorphism analysis of ORF3. Vaccine.

[10] Kweon C.H., Kwon B.J., Jung T.S., Kee Y.J., Hur D.H., Hwang E.K., Rhee J.C., An S.H. (1993). Isolation of porcine epidemic diarrhea virus (PEDV) in Korea. Korean J. Vet..

[11] Puranaveja S., Poolperm P., Lertwatcharasarakul P., Kesdaengsakonwut S., Boonsoongnern A., Urairong K., Kitikoon P., Choojai P., Kedkovid R., Teankum K. (2009). Chinese-like strain of porcine epidemic diarrhea virus, Thailand. Emerg. Infect. Dis..

[12] Takahashi K., Okada K., Ohshima K. (1983). An outbreak of swine diarrhea of a new-type associated with coronavirus-like particles in Japan. Nihon Juigaku Zasshi.

[13] Sun R.Q., Cai R.J., Chen Y.Q., Liang P.S., Chen D.K., Song C.X. (2012). Outbreak of porcine epidemic diarrhea in suckling piglets, China. Emerg. Infect. Dis..

[14] Li W., Li H., Liu Y., Pan Y., Deng F., Song Y., Tang X., He Q. (2012). New variants of porcine epidemic diarrhea virus, China, 2011. Emerg. Infect. Dis..

[15] Tian P.F., Jin Y.L., Xing G., Qv L.L., Huang Y.W., Zhou J.Y. (2014). Evidence of recombinant strains of porcine epidemic diarrhea virus, United States, 2013. Emerg. Infect. Dis..

[16] Stevenson G.W., Hoang H., Schwartz K.J., Burrough E.R., Sun D., Madson D., Cooper V.L., Pillatzki A., Gauger P., Schmitt B.J. (2013). Emergence of Porcine epidemic diarrhea virus in the United States: Clinical signs, lesions, and viral genomic sequences. J. Vet. Diagn. Investig..

[17] Fan B., Jiao D., Zhao X., Pang F., Xiao Q., Yu Z., Mao A., Guo R., Yuan W., Zhao P. (2017). Characterization of Chinese Porcine Epidemic Diarrhea Virus with Novel Insertions and Deletions in Genome. Sci. Rep..

[18] Jung K., Saif L.J., Wang Q. (2020). Porcine epidemic diarrhea virus (PEDV): An update on etiology, transmission, pathogenesis, and prevention and control. Virus Res..

[19] Guo J.H., Fang L.R., Ye X., Chen J.Y., Xu S.E., Zhu X.Y., Miao Y.M., Wang D., Xiao S.B. (2019). Evolutionary and genotypic analyses of global porcine epidemic diarrhea virus strains. Transbound. Emerg. Dis..

[20] Pensaert M.B., de Bouck P. (1978). A new coronavirus-like particle associated with diarrhea in swine. Arch. Virol..

[21] Song D., Moon H., Kang B. (2015). Porcine epidemic diarrhea: A review of current epidemiology and available vaccines. Clin. Exp. Vaccine Res..

[22] Chen Q., Li G.W., Stasko J., Thomas J.T., Stensland W.R., Pillatzki A.E., Gauger P.C., Schwartz K.J., Madson D., Yoon K.J. (2014). Isolation and Characterization of Porcine Epidemic Diarrhea Viruses Associated with the 2013 Disease Outbreak among Swine in the United States. J. Clin. Microbiol..

[23] Chang S.H., Bae J.L., Kang T.J., Kim J., Chung G.H., Lim C.W., Laude H., Yang M.S., Jang Y.S. (2002). Identification of the epitope region capable of inducing neutralizing antibodies against the porcine epidemic diarrhea virus. Mol. Cells.

[24] Gong L., Lin Y., Qin J., Li Q., Xue C., Cao Y. (2018). Neutralizing antibodies against porcine epidemic diarrhea virus block virus attachment and internalization. Virol. J..

[25] Thavorasak T., Chulanetra M., Glab-Ampai K., Mahasongkram K., Sae-Lim N., Teeranitayatarn K., Songserm T., Yodsheewan R., Nilubol D., Chaicumpa W. (2022). Enhancing epitope of PEDV spike protein. Front. Microbiol..

[26] Temeeyasen G., Srijangwad A., Tripipat T., Tipsombatboon P., Piriyapongsa J., Phoolcharoen W., Chuanasa T., Tantituvanont A., Nilubol D. (2014). Genetic diversity of ORF3 and spike genes of porcine epidemic diarrhea virus in Thailand. Infect. Genet. Evol..

[27] Thavorasak T., Chulanetra M., Glab-Ampai K., Teeranitayatarn K., Songserm T., Yodsheewan R., Sae-Lim N., Lekcharoensuk P., Sookrung N., Chaicumpa W. (2022). Novel Neutralizing Epitope of PEDV S1 Protein Identified by IgM Monoclonal Antibody. Viruses.

[28] Simon-Loriere E., Holmes E.C. (2011). Why do RNA viruses recombine?. Nat. Rev. Microbiol..

[29] Makino S., Keck J.G., Stohlman S.A., Lai M.M.C. (1986). High-Frequency Rna Recombination of Murine Coronaviruses. J. Virol..

[30] Jarvis M.C., Lam H.C., Zhang Y., Wang L.Y., Hesse R.A., Hause B.M., Vlasova A., Wang Q.H., Zhang J.Q., Nelson M.I. (2016). Genomic and evolutionary inferences between American and global strains of porcine epidemic diarrhea virus. Prev. Vet. Med..

[31] Wang H.N., Zhang L.B., Shang Y.B., Tan R.R., Ji M.X., Yue X.L., Wang N.N., Liu J., Wang C.H., Li Y.G. (2020). Emergence and evolution of highly pathogenic porcine epidemic diarrhea virus by natural recombination of a low pathogenic vaccine isolate and a highly pathogenic strain in the spike gene. Virus Evol..

[32] Dong N. (2017). Isolation, Identification and Serial Passaging Attenuation of Poricine Delta Coronavirus Strain CHN-HN-2014. Ph.D. Thesis.

[33] Song D.P., Huang D.Y., Peng Q., Huang T., Chen Y.J., Zhang T.S., Nie X.W., He H.J., Wang P., Liu Q.L. (2015). Molecular Characterization and Phylogenetic Analysis of Porcine Epidemic Diarrhea Viruses Associated with Outbreaks of Severe Diarrhea in Piglets in Jiangxi, China 2013. PLoS ONE.

[34] Katoh K., Standley D.M. (2013). MAFFT Multiple Sequence Alignment Software Version 7: Improvements in Performance and Usability. Mol. Biol. Evol..

[35] Nguyen L.T., Schmidt H.A., von Haeseler A., Minh B.Q. (2015). IQ-TREE: A fast and effective stochastic algorithm for estimating maximum-likelihood phylogenies. Mol. Biol. Evol..

[36] Lole K.S., Bollinger R.C., Paranjape R.S., Gadkari D., Kulkarni S.S., Novak N.G., Ingersoll R., Sheppard H.W., Ray S.C. (1999). Full-length human immunodeficiency virus type 1 genomes from subtype C-infected seroconverters in India, with evidence of intersubtype recombination. J. Virol..

[37] Zeng L. (2016). Proteome Analysis of PEDV-Infected Vero Cells and Attenution of PEDV by Serial Passage in Vero Cells. Ph.D. Thesis.

[38] Dong N., Fang L., Yang H., Liu H., Du T., Fang P., Wang D., Chen H., Xiao S. (2016). Isolation, genomic characterization, and pathogenicity of a Chinese porcine deltacoronavirus strain CHN-HN-2014. Vet. Microbiol..

[39] Holmes E.C. (2009). The Evolution and Emergence of RNA Viruses.

[40] Lee D.K., Park C.K., Kim S.H., Lee C. (2010). Heterogeneity in spike protein genes of porcine epidemic diarrhea viruses isolated in Korea. Virus Res..

[41] Chen Q., Gauger P.C., Stafne M.R., Thomas J.T., Madson D.M., Huang H.Y., Zheng Y., Li G.W., Zhang J.Q. (2016). Pathogenesis comparison between the United States porcine epidemic diarrhoea virus prototype and S-INDEL-variant strains in conventional neonatal piglets. J. Gen. Virol..

[42] Boniotti M.B., Papetti A., Lavazza A., Alborali G., Sozzi E., Chiapponi C., Faccini S., Bonilauri P., Cordioli P., Marthaler D. (2016). Porcine Epidemic Diarrhea Virus and Discovery of a Recombinant Swine Enteric Coronavirus, Italy. Emerg. Infect. Dis..

[43] Vlasova A.N., Marthaler D., Wang Q., Culhane M.R., Rossow K.D., Rovira A., Collins J., Saif L.J. (2014). Distinct characteristics and complex evolution of PEDV strains, North America, May 2013–February 2014. Emerg. Infect. Dis..

[44] Li R.F., Qiao S.L., Yang Y.Y., Guo J.Q., Xie S., Zhou E.M., Zhang G.P. (2016). Genome sequencing and analysis of a novel recombinant porcine epidemic diarrhea virus strain from Henan, China. Virus Genes.

[45] Wang X.W., Wang M., Zhan J., Liu Q.Y., Fang L.L., Zhao C.Y., Jiang P., Li Y.F., Bai J. (2020). Pathogenicity and immunogenicity of a new strain of porcine epidemic diarrhea virus containing a novel deletion in the N gene. Vet. Microbiol..

[46] Li X., Li Y., Huang J., Yao Y., Zhao W., Zhang Y., Qing J., Ren J., Yan Z., Wang Z. (2022). Isolation and oral immunogenicity assessment of porcine epidemic diarrhea virus NH-TA2020 strain: One of the predominant strains circulating in China from 2017 to 2021. Virol. Sin..

[47] Suzuki T., Shibahara T., Yamaguchi R., Nakade K., Yamamoto T., Miyazaki A., Ohashi S. (2016). Pig epidemic diarrhoea virus S gene variant with a large deletion non-lethal to colostrum-deprived newborn piglets. J. Gen. Virol..

[48] Li Z., Ma Z., Li Y., Gao S., Xiao S. (2020). Porcine epidemic diarrhea virus: Molecular mechanisms of attenuation and vaccines. Microb. Pathog..

[49] Deng X., van Geelen A., Buckley A.C., O’Brien A., Pillatzki A., Lager K.M., Faaberg K.S., Baker S.C. (2019). Coronavirus Endoribonuclease Activity in Porcine Epidemic Diarrhea Virus Suppresses Type I and Type III Interferon Responses. J. Virol..

[50] Hou Y., Ke H., Kim J., Yoo D., Su Y., Boley P., Chepngeno J., Vlasova A.N., Saif L.J., Wang Q. (2019). Engineering a Live Attenuated Porcine Epidemic Diarrhea Virus Vaccine Candidate via Inactivation of the Viral 2′-O-Methyltransferase and the Endocytosis Signal of the Spike Protein. J. Virol..

[51] Su S., Wong G., Shi W., Liu J., Lai A.C.K., Zhou J., Liu W., Bi Y., Gao G.F. (2016). Epidemiology, Genetic Recombination, and Pathogenesis of Coronaviruses. Trends Microbiol..

[52] Li W., van Kuppeveld F.J.M., He Q., Rottier P.J.M., Bosch B.J. (2016). Cellular entry of the porcine epidemic diarrhea virus. Virus Res..

[53] Hulswit R.J., de Haan C.A., Bosch B.J. (2016). Coronavirus Spike Protein and Tropism Changes. Adv. Virus Res..

