# Supplementary material for: The Characterization and Pathogenicity of a Recombinant Porcine Epidemic Diarrhea Virus Variant ECQ1
Source: Viruses. 2023 Jun 30;15(7):1492. doi: 10.3390/v15071492 (PMC10383920; doi:10.3390/v15071492)
Supplement: Supplementary file 1 [file viruses-15-01492-s001.zip › Supplementary Table S1.pdf]

Supplementary Table S1. Clinical samples information from 2017 to 2019

Year	Month	sample quantity	Place of samples collection (province)
2017	Jul.	34	Henan
	Aug.	15	Hubei. Henan
	Sept.	17	Hubei. Henan
	Oct.	13	Hubei
	Nov.	21	Hubei. Hunan
	Dec.	10	Hubei. Henan. Liaoning
2018	Jan.	23	Hunan
	Feb.	0	
	Mar.	45	Hubei. Henan. Hunan
	Apr.	36	Hunan
	May.	7	Hubei
	Jun.	57	Hubei. Hunan. Chongqing
	Jul.	52	Hubei. Hunan. Chongqing
	Aug.	46	Hunan
	Sept.	12	Sichuan
	Oct.	38	Hubei. Henan. Hunan. Jiangxi. Guangdong. Shanghai
	Nov.	44	Hubei. Henan. Fujian. Shandong. Guangdong. Hebei. Sichuan. Shanxi
	Dec.	51	Hubei. Henan. Guangdong. Guangxi. Hebei. Hainan. Guizhou. Fujian
2019	Jan.	30	Hubei. Henan. Guangdong. Hebei. Xinjiang. Sichuan. Chongqing
	Feb.	12	Hubei. Henan. Chongqing
	Mar.	51	Hubei. Henan. Fujian. Shandong. Liaoning. Heilongjiang. Hebei. Hunan. Sichuan. Jiangsu
	Apr.	11	Hubei. Henan. Hunan. Fujian
	May.	8	Hubei. Hunan
